# Pharmacodynamic Correlates of Linezolid Activity and Toxicity in Murine Models of Tuberculosis

**DOI:** 10.1093/infdis/jiaa016

**Published:** 2020-01-29

**Authors:** Kristina M Bigelow, Amelia N Deitchman, Si-Yang Li, Kala Barnes-Boyle, Sandeep Tyagi, Heena Soni, Kelly E Dooley, Rada M Savic, Eric L Nuermberger

**Affiliations:** 1 Department of Pharmacology and Molecular Sciences, Johns Hopkins University School of Medicine, Baltimore, Maryland, USA; 2 University of California San Francisco, Department of Bioengineering and Therapeutic Sciences, San Francisco, California, USA; 3 Center for Tuberculosis Research, Department of Medicine, Johns Hopkins University School of Medicine, Baltimore, Maryland, USA

**Keywords:** linezolid, mouse, pharmacodynamics, pharmacokinetics, tuberculosis

## Abstract

**Background:**

Linezolid (LZD) is bactericidal against *Mycobacterium tuberculosis*, but it has treatment-limiting toxicities. A better understanding of exposure-response relationships governing LZD efficacy and toxicity will inform dosing strategies. Because in vitro monotherapy studies yielded conflicting results, we explored LZD pharmacokinetic/pharmacodynamic (PK/PD) relationships in vivo against actively and nonactively multiplying bacteria, including in combination with pretomanid.

**Methods:**

Linezolid multidose pharmacokinetics were modeled in mice. Dose-fractionation studies were performed in acute (net bacterial growth) and chronic (no net growth) infection models. In acute models, LZD was administered alone or with bacteriostatic or bactericidal pretomanid doses. Correlations between PK/PD parameters and lung colony-forming units (CFUs) and complete blood counts were assessed.

**Results:**

Overall, time above minimum inhibitory concentration (T_>MIC_) correlated best with CFU decline. However, in growth-constrained models (ie, chronic infection, coadministration with pretomanid 50 mg/kg per day), area under the concentration-time curve over MIC (AUC/MIC) had similar explanatory power. Red blood cell counts correlated strongly with LZD minimum concentration (C_min_).

**Conclusions:**

Although T_>MIC_ was the most consistent correlate of efficacy, AUC/MIC was equally predictive when bacterial multiplication was constrained by host immunity or pretomanid. In effective combination regimens, administering the same total LZD dose less frequently may be equally effective and cause less C_min_-dependent toxicity.

## Teaser

Linezolid is an efficacious TB drug that exhibits dose- and duration-dependent toxicity. Using animal models, we show that decreasing dosing frequency while maintaining the same total weekly dose may be a viable strategy to maintain efficacy while decreasing toxicity.

Tuberculosis (TB) is the leading cause of death among infectious agents worldwide [1]. Multidrug-resistant (MDR) TB (resistant to isoniazid [INH] and rifampin [RIF]) threatens global TB control. Current treatment regimens for MDR-TB are 9–24 months in duration and have limited efficacy and substantial toxicity when used under field conditions. To achieve the World Health Organization goal of ending the TB epidemic by 2035 [1], new and repurposed agents must be developed and optimized to create regimens that are shorter, safer, and more effective.

Linezolid (LZD) is a licensed oxazolidinone antibiotic repurposed for treatment of TB. Initially reserved for salvage situations, it was recently elevated to Group A (medicines prioritized for inclusion in regimens) in evidence-based guidelines for MDR-TB treatment [2]. Linezolid is also part of a novel, 6-month oral regimen containing bedaquiline and pretomanid that recently demonstrated promising efficacy in patients with extensively drug-resistant (XDR) TB (MDR-TB with additional resistance to fluoroquinolones and injectables) in the Phase 3 Nix-TB trial [3]. However, LZD causes toxicity related to both dose and duration that is often treatment-limiting [3–20]. Bone marrow toxicity (including anemia and thrombocytopenia) typically occurs within the first 1–2 months of LZD treatment with doses ≥600 mg/day, whereas peripheral and more rare optic neuropathies occur with longer treatment durations despite lowering the dose to 300 mg/day [9, 14]. Despite its new status as a Group A drug, the optimal LZD-dosing strategies that best balance its efficacy with risk of toxicity remain uncertain.

A thorough understanding of exposure-response relationships governing LZD efficacy and toxicity will inform optimal dosing strategies. Although recent studies have advanced the state of knowledge, the results have been inconclusive. Linezolid toxicity is caused by inhibition of mitochondrial protein synthesis (MPS). Several clinical studies suggest that LZD toxicity correlates best with minimum concentrations (C_min_) [21–24]. However, studies using in vitro hollow fiber systems using mitochondrial protein content or gene expression as surrogate toxicity markers yielded differing results, identifying either C_min_ [25] or area under the concentration-time curve (AUC) [26] as the pharmacokinetic/toxicodynamic (PK/TD) parameter best correlated with MPS inhibition. Likewise, studies using in vitro hollow fiber systems to assess pharmacokinetic/pharmacodynamic (PK/PD) relationships alternately identified time above minimum inhibitory concentration (T_>MIC_) [25, 27] or AUC/MIC [26] as the PK/PD parameter most strongly correlated with LZD activity against *Mycobacterium tuberculosis*. In vivo studies assessing PK/PD relationships are limited [28]. In an effort to reconcile seemingly conflicting data [25–28], we hypothesized that the PK/PD parameters best correlated with LZD efficacy depend on the net bacterial multiplication rate, which is itself a function of immune pressure and effects of companion agents. We set out to study PK/PD and PK/TD relationships more comprehensively under in vivo conditions and to address the influence of bacterial multiplication rate on the PK/PD correlates of LZD activity.

## METHODS

### Mycobacterial Strain

Experiments used *M tuberculosis* H37Rv. The LZD MIC is 1 µg/mL using the broth macrodilution method in Middlebrook 7H9 medium (Thermo Fisher Scientific, Pittsburgh, PA). Cultures were grown in 7H9 broth supplemented with 10% oleic acid-albumin-dextrose-catalase ([OADC] Difco Laboratories, Detroit, MI) and 0.05% Tween 80 (Sigma-Aldrich, St. Louis, MO) before infection.

### Antimicrobials

Isoniazid and RIF (Sigma-Aldrich) were dissolved in distilled water. Pretomanid and LZD were provided by the Global Alliance for TB Drug Development. Pretomanid was prepared in the CM-2 (cyclodextrin micelle) formulation, and LZD was suspended in 0.5% methylcellulose solution. Dosing formulations were prepared weekly and stored at 4°C as described previously [29, 30].

### Pharmacokinetics of Linezolid in Mice

All procedures involving animals were approved by the Animal Care and Use Committee of Johns Hopkins University. Multidose PK of LZD in plasma was characterized in uninfected female BALB/c mice (Charles River Laboratories, Wilmington, MA) receiving oral doses of 10, 30, 100, or 335 mg/kg once daily. After 5 days of dosing, 3 mice per group per time point were sampled by submandibular bleed at 0, 0.5, and 1 hour or at 0, 0.5, and 8 hours postdose and then sacrificed and sampled by cardiac puncture at 4 or 24 hours, respectively. Linezolid was quantified by a validated high-performance liquid chromatography method (Infectious Disease Pharmacokinetics Laboratory, University of Florida, Gainesville, FL) [31]. Concentration-time data were analyzed initially by standard noncompartmental techniques using WinNonlin (version 7.0; Pharsight, Mountain View, CA).

### Population Pharmacokinetic Modeling of Linezolid in Mice

A population-based, nonlinear, mixed-effects modeling approach (in NONMEM version 7.4) was used to develop a PK model to describe mouse PK data. The final structural model (eg, 1- versus 2-compartment models), linear versus nonlinear absorption and/or elimination, and residual error model (additive, proportional, or combined) was selected based on goodness-of-fit plots, visual predictive checks, precision of model parameter estimates, and relative change in objective function value.

### Aerosol Mouse Infection Models

Using an inhalation exposure system (Glas-Col, Terre Haute, IN), 6-week-old female BALB/c mice were infected with a log-phase culture of *M tuberculosis* (optical density at 600 nm of approximately 1.0). After infection, mice were randomized into treatment groups (3 mice per group). Untreated mice were sacrificed (1) the day after infection to determine colony-forming unit (CFU) implantation in the lungs, (2) at initiation of treatment to determine pretreatment CFU counts, and (3) 28 days postinfection to count CFU in untreated controls. Two infection models were used to vary the bacterial growth state before LZD treatment: a log phase growth (acute infection) model (performed twice) and a no-net-growth (chronic infection) model. In the acute infection model, mice were infected with approximately 4 log_10_ CFU and dosing started 7 days later. In the chronic infection model, in which the immune system suppresses bacterial growth, mice were infected with approximately 2 log_10_ CFU and dosing started 28 days later. To complement LZD monotherapy experiments in acute and chronic infection models, coadministration of pretomanid was also used to modulate the bacterial growth rate in the acute infection model. In these experiments, LZD was coadministered with 1 of 2 doses of pretomanid—12.5 mg/kg per day or 50 mg/kg per day. These pretomanid doses provided for a slowed-growth model and a no-net-growth model, respectively.

### Linezolid Dose Fractionation and Study Treatment

All drugs except LZD were administered 5 days per week (5 of 7). Linezolid was administered 3, 5, or 7 days per week. All drugs were given by gavage. Pretomanid was given at least 4 hours before LZD [32]. Three total (cumulative) weekly doses of LZD were used in dose-fractionation experiments: 100, 300, and 1000 mg/kg per week. Each total weekly dose was fractionated up to 5 ways: twice-daily (BID) 7 of 7, BID 5 of 7, once-daily (QD) 7 of 7, QD 5 of 7, and QD thrice-weekly (3 of 7). Controls included no treatment, LZD 100 mg/kg (5 of 7) (which produces a plasma AUC similar to the average plasma AUC after a 1200-mg dose in humans) [33], INH, and RIF, each at 10 mg/kg (5 of 7). One-way analysis of variance (ANOVA) with Tukey’s posttest was used to compare group means within each total weekly dose level.

### Assessment of Treatment Efficacy

Lung CFU counts were assessed at the onset of treatment and after 28 days of treatment by performing quantitative cultures of lung homogenates on OADC-enriched 7H11 agar (Difco Laboratories) as previously described [34, 35].

### Pharmacokinetics/Pharmacodynamics Analysis

The population PK model developed in NONMEM was used to simulate concentration-time profiles and estimate PK/PD parameters for dosing regimens tested in dose-fractionation studies. Relationships between log_10_-transformed PK/PD parameters (AUC/MIC, T_>MIC_, and C_max_/MIC) and log_10_-transformed change in CFU counts compared with untreated controls (LZD monotherapy experiments) or pretomanid-treated controls (LZD-pretomanid combination experiments) were assessed. Correlation analysis was performed using an inhibitory sigmoid E_max_ model with variable slope to describe the relationship between each PK/PD parameter and the change in lung CFU count versus controls not receiving LZD. R-squared values were used to evaluate goodness-of-fit. All analyses were performed with Prism v.6.01 (GraphPad Software, San Diego, CA).

### Assessment of Treatment Toxicity

Whole blood was collected from a separate cohort of infected mice treated for 8 weeks alongside a dose-fractionation study in the acute infection model and sent for complete blood count (CBC) analysis. One-way ANOVA with Tukey’s posttest was used to compare group means within each total weekly dose level.

### Pharmacokinetic/Toxicodynamic Analysis

Correlation analysis was performed using an inhibitory sigmoid E_max_ model with variable slope to describe the relationship between each log_10_-transformed PK/TD parameter (AUC, C_max_, or C_min_) derived from PK simulations and various white blood cell, red blood cell, and platelet measures. R-squared values were used to evaluate goodness-of-fit.

## RESULTS

### Linezolid Pharmacokinetic in Mice

**Figure 1.**
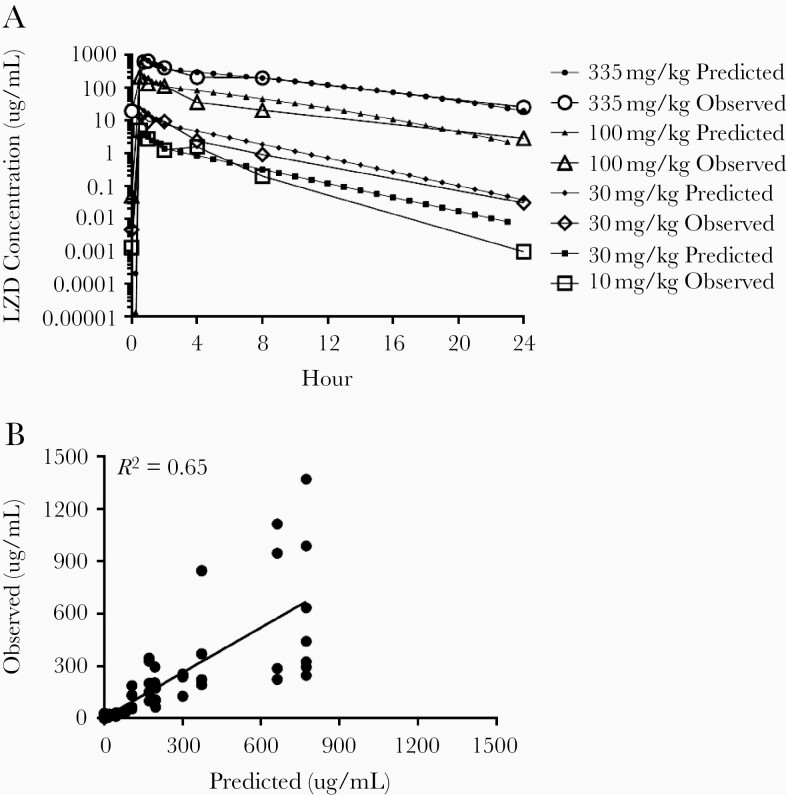
(A) Observed versus model-predicted linezolid (LZD) concentration-time curves. (B) Observed versus model-predicted LZD concentrations and regression line (y = 0.86x + 5.6).

Pharmacokinetic model parameter estimates from noncompartmental analyses are shown in Supplemental Table 1. Because the relationship between dose and exposure was nonlinear at higher doses, we developed a population PK model describing LZD PK in mice. A 2-compartment structural model with increasing bioavailability with dose, separate saturable (Michaelis-Menten), and linear clearance pathways, and proportional residual error model provided the best model fit, with precise parameter estimates (Table 1). Figure 1A shows comparisons of the observed PK data compared with the model-predicted data. The observed versus predicted concentrations are shown for all doses (Figure 1B).

**Table 1.** Population PK Parameter Estimates

Model Parameter Estimates	
Parameter (Units)	Estimate (RSE, %)
V_MAX_ (mg/h per kg)	3.26 (18)
CL_in_ (L/h per kg)	0.0649 (16)
V_C_ (L/kg)	0.268 (17)
k_a_ (h^−1^)	7.32 (4)
K_M_ (mg/L)	26.4 FIX
Q (L/h per kg)	0.504 (20)
V_P_ (L/kg)	0.402 (14)
F_10_ mg/kg	0.184 (5)
F_30_ mg/kg	0.233 (9)
F_100_ mg/kg	1 FIX
F_335_ mg/kg	1 FIX
Proportional variability	0.796 (8)

Abbreviations: CL_in_, intrinsic clearance;  F, bioavailability; k_a_, absorption rate constant; K_M_, Michaelis-Menten constant; PK, pharmacokinetic; Q, blood flow rate; RSE, relative standard error; V_C_, total body volume of distribution; V_MAX_, maximum rate of metabolism; V_P_, plasma volume of distribution.

### Efficacy of Linezolid in Dose-Fractionation Studies

**Figure 2.**
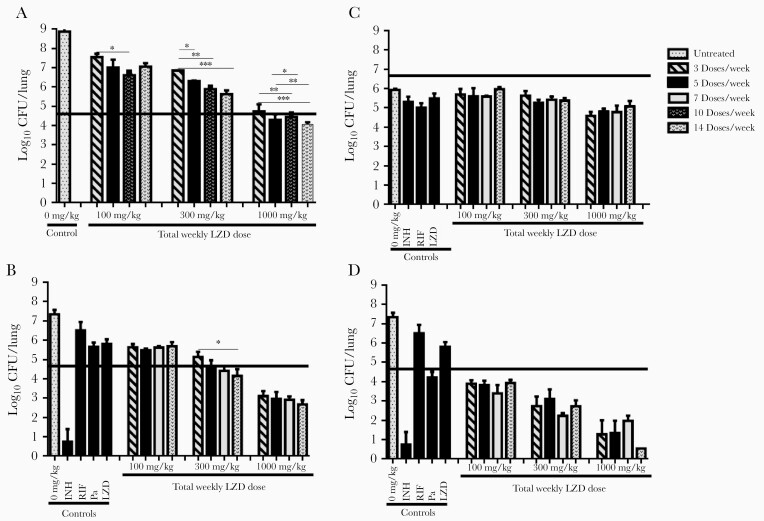
Mean lung colony-forming unit (CFU) counts (±standard deviation) in BALB/c mice after 28 days of treatment with linezolid (LZD) alone or in combination with pretomanid (Pa) in dose-fractionation studies. (A) Linezolid alone in acute infection model. (B) Linezolid in combination with Pa 12.5 mg/kg in acute infection model. (C) Linezolid alone in chronic infection model. (D) Linezolid in combination with Pa 50 mg/kg in acute infection model. Four total weekly LZD doses (0, 100, 300, and 1000 mg/kg per week) were fractionated into 4 dosing schedules: 3 doses/week, 5 doses/week, either 7 or 10 doses/week, and 14 doses/week. The isoniazid (INH), rifampin (RIF), and LZD controls received commonly used doses of 10, 10, and 100 mg/kg per day, respectively, 5 doses/week. Thick horizontal lines indicate the mean CFU count at treatment initiation. Statistical significance determined using one-way analysis of variance with Tukey’s posttest to adjust for multiple comparisons within each dose level: *, *P* < .05; **, *P* < .01; ***, *P* < .001.

As expected for the acute infection model, CFU counts were increasing logarithmically in the lungs at the start (Day 0) of LZD monotherapy (Figure 2A). At Day 0, the mean lung CFU count (±standard deviation) was 4.35 ± 0.24 log_10_. By the end of the treatment period (Day 28), the mean CFU count in untreated controls was 8.35 ± 0.61 log_10_. As expected, LZD effects increased with total weekly dose. However, it was plainly evident that, at a given total weekly dose, LZD effects increased with increasing dosing frequency (Figure 2A and Supplemental Table 2). Although the experiments were not powered to test for significant differences between dosing schedules at each weekly dose level, groups in net-growth models receiving more frequent dosing of the same total weekly dose often had statistically significantly lower lung CFU counts than groups receiving less frequent dosing. These results indicate that, when the total weekly dose is fixed, increasing the dosing frequency results in greater LZD effects against multiplying bacterial populations. The results shown in Figure 2A are representative of 2 experiments performed in the acute infection model.

Similar findings were observed in the acute infection model in which pretomanid 12.5 mg/kg was used to slow the multiplication rate. The Day 0 mean log_10_ CFU count was 4.75 ± 0.18. By Day 28, the mean log_10_ CFU count in untreated mice was 7.33 ± 0.19. Low-dose pretomanid alone inhibited growth but did not prevent a net increase in the mean lung log_10_ CFU count, which reached 5.66 ± 0.18 at Day 28 (*P* < .01 vs Day 0). As with LZD alone in the acute infection model, LZD coadministered with low-dose pretomanid resulted in increasing effect with increasing total weekly dose, and, at each active weekly dose level, more frequent dosing increased LZD effect (Figure 2B and Supplemental Table 2). These data suggest that when LZD is combined with weak companion agents, its activity is time-dependent.

When growth was suppressed by host immunity in the chronic infection model, the magnitude of LZD effects was more limited, and more frequent administration of a given total weekly dose did not increase LZD effect (Figure 2C and Supplemental Table 2). Similar findings were observed in the acute infection model with coadministration of the fully bacteriostatic pretomanid dose of 50 mg/kg per day (*P* = .41 vs Day 0). Increasing total weekly LZD doses yielded increasing bactericidal effects, but there was no apparent benefit to more frequent dosing schedules at a given total weekly dose level (Figure 2D and Supplemental Table 2).

### Pharmacokinetics/Pharmacodynamics Analysis

**Figure 3.**
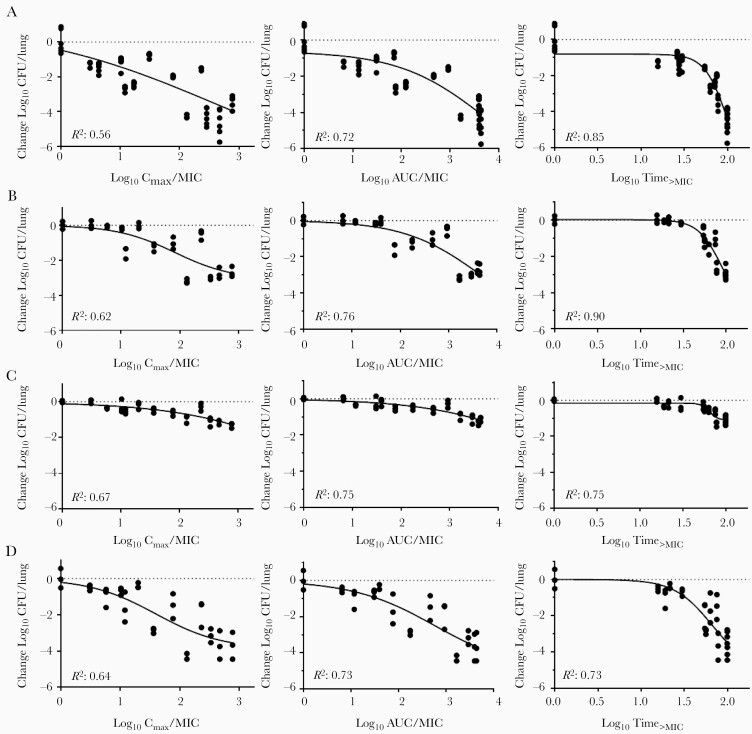
Relationships between linezolid (LZD) pharmacokinetic/pharmacodynamics parameters and change in lung colony-forming unit (CFU) counts in each infection model. (A) Linezolid monotherapy in acute infection model. (B) Linezolid in combination with pretomanid at 12.5 mg/kg in acute infection model. (C) Linezolid monotherapy in chronic infection model. (D) Linezolid in combination with pretomanid at 50 mg/kg in acute infection model. Dotted horizontal line indicates no change from CFU count in untreated controls (A and C) or no change from CFU count after treatment with pretomanid alone (B and D). AUC/MIC, area under the concentration-time curve above the minimum inhibitory concentration; C_max_, LZD maximum concentration; Time_>MIC_, time above minimum inhibitory concentration.

Population PK models were used to simulate all doses used in the dose-fractionation experiments. A linear equation was used to estimate increasing bioavailability for doses between 30 and 100 mg/kg (F = 0.233 + 0.0110 × (Dose-30)). Secondary plasma PK/PD parameters (AUC/MIC, C_max_/MIC, and T_>MIC_) were estimated. The PK/PD modeling was performed to evaluate their relationships with microbiologic outcomes. For LZD monotherapy in the acute infection model, activity correlated best with T_>MIC_ (R^2^ = 0.85), followed by AUC/MIC (R^2^ = 0.72) (Figure 3A). Likewise, when LZD was combined with low-dose pretomanid, which did not fully suppress multiplication, activity again correlated best with T_>MIC_ (R^2^ = 0.90), followed by AUC/MIC (R^2^ = 0.76) (Figure 3B). In contrast, when the infection model allowed no net growth in the absence of LZD treatment, LZD activity correlated just as well with AUC/MIC compared with T_>MIC_ (Figure 3C and D). Therefore, under conditions of no net growth, the LZD activity is no longer as dependent on T_>MIC_.

### Pharmacokinetic/Toxicodynamic Analysis

**Figure 4.**
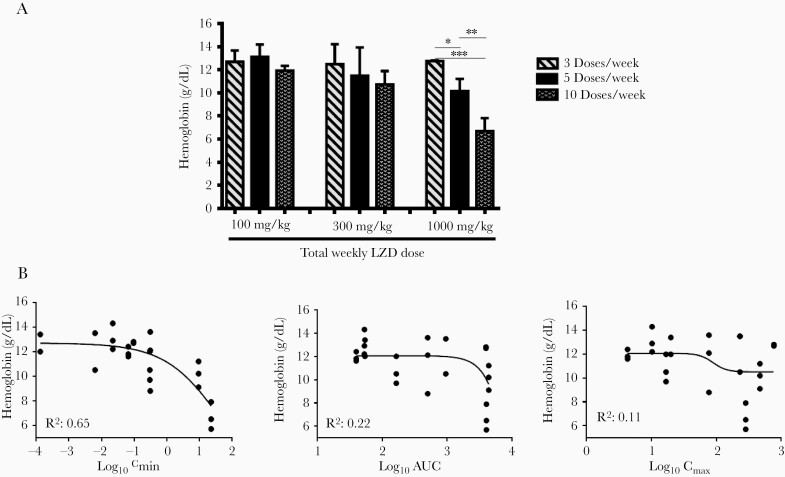
Exposure-response relationship for linezolid (LZD)-induced anemia, as measured by hemoglobin in infected BALB/c mice. (A) Mean hemoglobin by total weekly dose and dosing frequency. (B) Relationships between pharmacokinetic/toxicodynamic parameters and hemoglobin. Three total weekly LZD doses (0, 100, 300, and 1000 mg/kg per week) were fractionated into 3 dosing schedules: 3 doses/week, 5 doses/week, and 10 doses/week. Statistical significance determined using one-way analysis of variance with Tukey’s posttest to adjust for multiple comparisons within each dose level: *, *P* < .05; **, *P* < .01; ***, *P* < .001. AUC, area under the concentration-time curve; C_max_, LZD maximum concentration; C_min_, LZD minimum concentration.

The population PK model was used to simulate all doses used in the toxicity substudy and generate secondary PK/TD parameter estimates for AUC, C_max_, and C_min_. Relationships between these parameters and various cell count measures from the CBC were assessed. Although no significant relationship was observed between LZD exposure and white blood cell or platelet counts (Supplemental Table 3), increasing total weekly LZD dose was associated with decreasing red blood cell indices, including hemoglobin concentration (Figure 4A). The change in hemoglobin concentration was correlated with LZD C_min_ (R^2^ = 0.65) but not with AUC or C_max_ (Figure 4B). Similar observations were made using hematocrit and red blood cell counts (Supplemental Figure 1). The C_min_ value associated with a reduction in hemoglobin to 8 g/dL was 27.5 μg/mL (Figure 4B).

## DISCUSSION

Linezolid has a narrow therapeutic margin [36]. Despite its new position among preferred agents for treatment of MDR/XDR-TB [2], the optimal LZD-dosing strategy that maximizes efficacy while minimizing toxicity remains undefined and may depend on the clinical setting [37]. Thorough understanding of exposure-response relationships governing its anti-TB activity and toxicity should inform LZD-dosing strategies.

Studies with LZD in in vitro hollow fiber TB models alternately identified T_>MIC_ and AUC/MIC as being most strongly associated with microbial kill. Brown et al [25] identified T_>MIC_ in a model in which *M tuberculosis* was multiplying logarithmically in the absence of any constraints, whereas Deshpande et al [38] identified AUC/MIC in a model in which *M tuberculosis* was contained in activated THP-1 macrophages, and Srivastava et al [26] identified AUC/MIC in a model in which *M tuberculosis* multiplication was constrained by acidified media. Limited in vivo data from a mouse model in which LZD or sutezolid treatment was timed with the onset of the adaptive immune response also suggested that efficacy was linked to AUC/MIC [28]. Taking these prior studies into account, we hypothesized that the PK/PD parameter most closely linked to the LZD effect may vary based, at least in part, on the net multiplication of *M tuberculosis* in the model system under study, and we set out to assess LZD PK/PD relationships in mouse models with varying growth constraints. The results presented here indicate that without growth suppression by the immune response or an effective, adequately dosed companion agent, LZD activity is time-dependent and correlates best with T_>MIC_. However, when net bacterial multiplication is completely suppressed by acquired immune responses or strong companion agents, T_>MIC_ is no longer the single PK/PD parameter most associated with LZD effect and AUC/MIC becomes equally important. These findings may help to explain the mixed results from previous in vitro studies [25–27, 38] and inform strategies for optimal clinical usage of LZD. For example, in the first few weeks of TB treatment, when net bacterial multiplication is highest, the optimal LZD-dosing regimen is likely to maximize T_>MIC_, especially for patients with large bacillary burdens and/or severe immunodeficiency and those treated with weak companion drugs. However, as net multiplication approaches zero over time and/or in selected lesion compartments through the effects of the acquired immune response and actions of companion drugs, the need to maximize T_>MIC_ diminishes and enables more intermittent LZD administration that may be better tolerated without sacrificing efficacy.

The reduced time dependence of LZD’s anti-TB effect under certain in vivo conditions demonstrated here affords some flexibility in selecting dosing schedules to minimize its dose- and duration-dependent toxicities. These common, often treatment-limiting hematopoietic and neuropathic toxicities [9] are attributed to MPS inhibition and reductions in proteins critical to cellular respiration [25]. These toxic effects are difficult to fully divorce from the antimicrobial effects given the similar ontogeny of bacterial and mitochondrial ribosome targets. However, the preponderance of data from an in vitro hollow fiber toxicity model and clinical observations suggests that LZD toxicity is governed by trough concentrations (C_min_) that exceed cell-specific thresholds for MPS inhibition [21–25]. Consistent with these results, we identified C_min_ as the parameter most closely correlated with LZD-induced anemia in mice. Although ours is not the first study to describe LZD-induced anemia in mice [39, 40], it is the first to show that anemia is linked to LZD C_min_. Previous studies evaluating toxicity of LZD or chloramphenicol, which causes myelotoxicity through a similar mechanism [41], also found that anemia is a more common manifestation of mitochondrial toxicity than thrombocytopenia in rodents [39, 40, 42]. Although thrombocytopenia is more common when LZD is used clinically to treat acute bacterial infections [22, 43], we note that anemia was a more frequent treatment-limiting toxicity among XDR-TB patients in the Nix-TB trial [3], perhaps because platelets are an acute phase reactant that are often elevated in chronic infections such as TB, whereas red blood cell counts are often depressed at baseline. Considering the potential clinical relevance of our PK/TD results, we note that the C_min_ threshold associated with a blood hemoglobin concentration below 8 mg/dL, the threshold for grade 3 clinical toxicity, was 27.5 µg/mL, a value higher than, but not too dissimilar to, threshold values for thrombocytopenia identified in previous human studies in the range of 6.5–9.3 µg/mL [21–24]. Other inbred mouse strains such as C3H and CBA appear more susceptible to drug-induced hematological toxicity and are susceptible to *M tuberculosis* infection [42–45]. Therefore, they could be even more attractive as models for oxazolidinone TD studies. Our findings provide further support for LZD C_min_ as a predictive parameter for hematologic toxicity and indicate that mice may be useful for evaluating the TDs of other oxazolidinones being considered as TB drugs, including new agents with reduced MPS inhibition, designed to increase the therapeutic margin [37].

In our study, the multidose PK of LZD in mice was best described by a model with dose-dependent bioavailability and saturable clearance resulting in supraproportional increases in LZD exposure with increasing dose size. Reduced LZD clearance with increasing dose and duration of administration in humans is well described [44, 45], although perhaps underappreciated by clinicians. Indeed, it has been proposed that LZD inhibits its own metabolism with repeated administration of doses sufficient to inhibit MPS [45]. One implication of time-dependent saturable clearance of LZD is that C_min_ will increase over time without a change in dose, which may increase the risk of mitochondrial toxicity. Thus, the expected dose- and time-dependent changes in LZD clearance over time further support a strategy of transitioning to more intermittent dosing of LZD over time on treatment to reduce the risk of toxicity.

The in vivo PK/PD and PK/TD relationships described here support the use of more intermittent dosing of LZD (eg, dividing the same weekly dose into thrice weekly or every other day) under certain conditions to preserve drug efficacy while minimizing C_min_-driven toxicity. Specifically, although doses achieving higher T_>MIC_ (eg, 900–1200 mg daily) may optimize bacterial kill during the first few weeks of treatment, including killing of mutant subpopulations resistant to companion agents, a transition to 600–1200 mg every other day or thrice weekly may offer similar anti-TB activity but reduced toxicity when compared with 300–600 mg daily among patients with adequate immune responses receiving strong companion agents. Achieving higher LZD peaks with intermittent 1200-mg doses may also reduce the selection of LZD-resistant mutants [46]. Ongoing clinical trials with LZD dose fractionation should further inform the optimal LZD dose and dosing schedule. The LZD dose administered with pretomanid and bedaquiline changed from 600 mg twice daily to 1200 mg once daily midway during the Nix-TB trial (ClinicalTrials.gov Identifier: NCT02333799). The TB-PRACTECAL trial (ClinicalTrials.gov Identifier: NCT02589782) is comparing a LZD 300 mg daily to 600 mg thrice weekly after the first 16 weeks of treatment. Our findings presented here suggest that more frequent LZD dosing is not necessary when it is given with potent companion drugs or later in the course of treatment and that less frequent dosing is safer. Results from these clinical trials will be helpful for determining clinical efficacy and toxicity thresholds in effective multidrug regimens.

## CONCLUSIONS

Our study has limitations. BALB/c mouse TB models produce only cellular lung granulomas, and virtually all infecting bacilli reside intracellularly. In contrast, the hallmarks of human TB pathology are caseating lung lesions and cavities [47]. Although the BALB/c mouse model is tractable, yields reproducible results, and provides a good starting place for assessing in vivo PK/PD relationships [48], C3HeB/FeJ mouse TB models produce caseating lung pathology that enables study of drug distribution into such lesions and activity against large extracellular bacterial populations in caseum [49, 50]. Experiments in C3HeB/FeJ mice to confirm our findings in BALB/c mice are underway. Another caveat is that the time course of LZD concentrations in mice does not precisely mimic human concentration-time curves. However, this concern should be largely mitigated by our dose-fractionation methodology and the PK/PD models accounting for observed PK.

## Supplementary Data

Supplementary materials are available at *The Journal of Infectious Diseases* online. Consisting of data provided by the authors to benefit the reader, the posted materials are not copyedited and are the sole responsibility of the authors, so questions or comments should be addressed to the corresponding author.

jiaa016_suppl_Supplementary_Figure_1

jiaa016_suppl_Supplementary_Tables_1-3

## Notes

***Financial support.*** This work was funded by the National Institutes of Health (R01-AI-111992) and the Global Alliance for TB Drug Development. A. N. D. was funded by National Institutes of Health Grants T32GM007546, R01AI111992, R01AI111967, and R01AI106398.

***Potential conflicts of interest.*** E. L. N. is an inventor on patent US20180280401A1 (pending). All authors have submitted the ICMJE Form for Disclosure of Potential Conflicts of Interest. Conflicts that the editors consider relevant to the content of the manuscript have been disclosed.

Presented in part: American Society for Microbiology Microbe 2017, June 1–5, 2017, New Orleans LA; 10th International Workshop on Clinical Pharmacology of Tuberculosis Drugs, October 15, 2017, Atlanta GA; American Society for Microbiology Microbe 2018, June 6–11, 2018, Atlanta GA; 11th International Workshop on Clinical Pharmacology of Tuberculosis Drugs, October 23, 2018, The Hague, Netherlands.
