# Supplementary figures and images for: Pharmacodynamic Correlates of Linezolid Activity and Toxicity in Murine Models of Tuberculosis
Source: J Infect Dis. 2020 Jan 29;223(11):1855–64. doi: 10.1093/infdis/jiaa016 (PMC8176636; doi:10.1093/infdis/jiaa016)

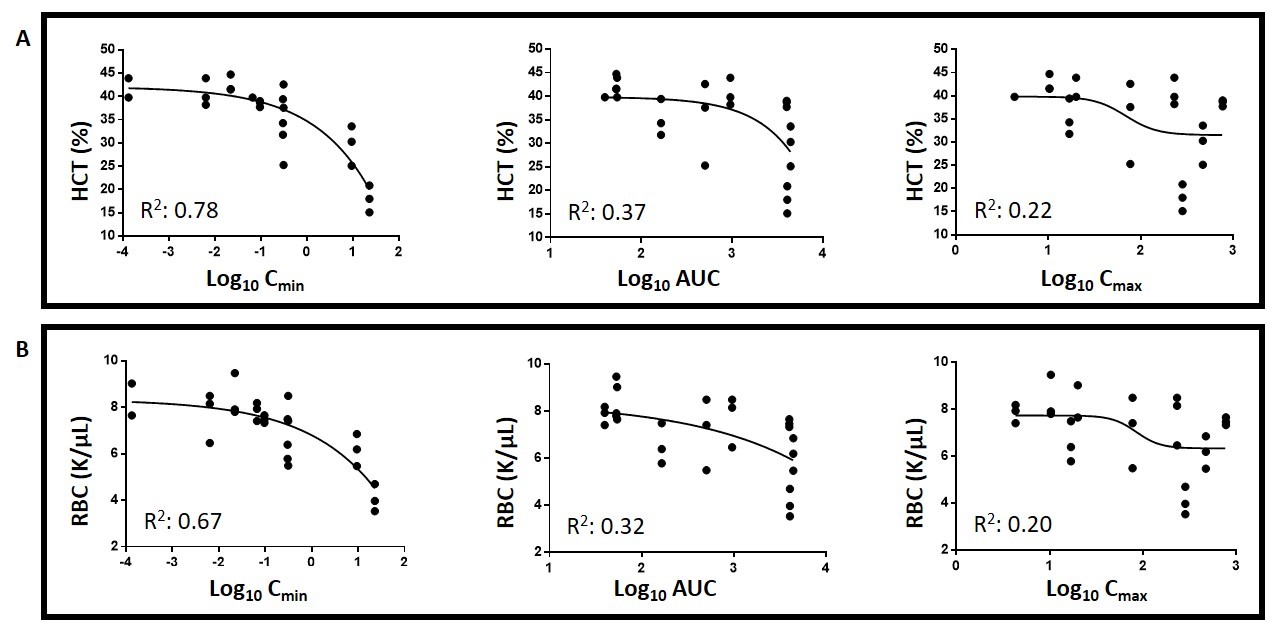

Supplement: jiaa016_suppl_Supplementary_Figure_1 [file jiaa016_suppl_supplementary_figure_1.jpeg]
