# Supplementary material for: Pharmacodynamic Correlates of Linezolid Activity and Toxicity in Murine Models of Tuberculosis
Source: J Infect Dis. 2020 Jan 29;223(11):1855–64. doi: 10.1093/infdis/jiaa016 (PMC8176636; doi:10.1093/infdis/jiaa016)
Supplement: jiaa016_suppl_Supplementary_Tables_1-3 [file jiaa016_suppl_supplementary_tables_1-3.docx]

**SUPPLEMENTARY TABLES AND FIGURE LEGENDS**

**Supplemental Table 1.** PK parameter estimates from noncompartmental analysis of LZD doses 10, 30, 100 and 335 mg/kg/day after 5 days of treatment. Abbreviations: LZD, linezolid; AUC_0-24_, area under the concentration-time curve from point zero to 24 h after drug intake; C_max_, LZD maximum drug concentration; C_min_, LZD minimum concentration; T_max_, time to reach maximum LZD concentration; t_1/2_, half-life of LZD; Cl_ss_/F, total body clearance at steady state following oral administration; V_z_/F, apparent volume of distribution following oral administration.

|  | **Dose** | | | |
| --- | --- | --- | --- | --- |
| **Parameter** | **10 mg/kg** | **30 mg/kg** | **100 mg/kg** | **335 mg/kg** |
| **AUC_0-24_ (mg*h/L)** | 12.62 | 42.09 | 791.27 | 4898.45 |
| **C_max_ (mg/L)** | 6.17 | 19.28 | 346.90 | 847.25 |
| **C_min_ (mg/L)** | 0.00 | 0.01 | 0.38 | 25.22 |
| **T_max_ (h)** | 0.50 | 0.55 | 0.50 | 1.47 |
| **t_1/2_ (h)** | 2.64 | 2.69 | 3.57 | 5.64 |
| **Cl_ss_/F (L/h)** | 0.02 | 0.01 | 0.00 | 0.00 |
| **V_z_/F (L)** | 0.06 | 0.06 | 0.01 | 0.01 |

**Supplemental Table 2**. Mean lung log_10_ CFU counts (± SD) in BALB/c mice at the initiation of treatment (Day 0) and after 28 days of treatment with the indicated regimen. Abbreviations: LZD, linezolid; Pa, pretomanid; INH, isoniazid; RIF, rifampin; qd, once a day dosing; qod, every other day dosing (M-W-F); bid, twice a day dosing.

|  |  | **Models with net bacterial multiplication** | | **Models with no net bacterial multiplication** | |
| --- | --- | --- | --- | --- | --- |
| **Total LZD dose per week** | **Control or LZD dosing schedule** | **LZD monotherapy in acute infection model** | **LZD combined with Pa 12.5 mg/kg in acute infection model** | **LZD monotherapy in chronic infection model** | **LZD combined with Pa 50 mg/kg in acute infection model** |
| **Controls** | Day 0 | 4.35±0.24 | 4.75±0.18 | 6.82 ± 0.06 | 4.75±0.18 |
|  | Untreated (Day 28) | 8.35±0.61 | 7.33±0.19 | 6.33 ± 0.05 | 7.33±0.19 |
|  | INH (5/7) qd |  | 0.74±0.54 | 5.31±0.22 | 0.74±0.54 |
|  | RIF (5/7) qd |  | 6.51±0.35 | 5.00±0.19 | 6.51±0.35 |
|  | Pa 12.5 mg/kg (5/7) qd |  | 5.66±0.18 |  |  |
|  | Pa 50 mg/kg (5/7) qd |  |  |  | 4.45±0.44 |
|  | LZD 100 mg/kg (5/7) qd |  | 5.80±0.20 | 5.49±0.21 | 5.80±0.20 |
| **LZD 100 mg/kg** | LZD 33 mg/kg (3/7) qod | 7.60±0.11 | 5.63±0.15 | 6.13±0.17 | 4.04±0.12 |
|  | LZD 20 mg/kg (5/7) qd | 7.19±0.30 | 5.49±0.06 | 6.06±0.30 | 3.74±0.16 |
|  | LZD 14 mg/kg (7/7) qd |  | 5.61±0.06 | 5.98±0.04 | 3.46±0.44 |
|  | LZD 10 mg/kg (5/7) bid | 6.82±0.25 |  |  |  |
|  | LZD 7 mg/kg (7/7) bid | 7.05±0.15 | 5.68±0.18 | 6.36±0.09 | 3.93±0.12 |
| **LZD 300 mg/kg** | LZD 100 mg/kg (3/7) qod | 6.80±0.07 | 5.12±0.23 | 6.04±0.18 | 2.60±0.60 |
|  | LZD 60 mg/kg (5/7) qd | 6.34±0.05 | 4.62±0.28 | 5.71±0.13 | 2.94±0.56 |
|  | LZD 43 mg/kg (7/7) qd |  | 4.41±0.19 | 5.85±0.12 | 1.58±0.12 |
|  | LZD 30 mg/kg (5/7) bid | 5.93±0.12 |  |  |  |
|  | LZD 21 mg/kg (7/7) bid | 5.64±0.15 | 4.13±0.28 | 5.79±0.11 | 2.82±0.68 |
| **LZD 1000 mg/kg** | LZD 333 mg/kg (3/7) bid | 4.82±0.35 | 2.98±0.24 | 5.01±0.12 | 0.75±0.60 |
|  | LZD 200 mg/kg (5/7) qd | 3.70±0.68 | 2.90±0.26 | 5.20±0.11 | 0.76±0.64 |
|  | LZD 143 mg/kg (7/7) qd |  | 2.65±0.08 | 5.22±0.21 | 1.28±0.31 |
|  | LZD 100 mg/kg (5/7) bid | 4.07±0.43 |  |  |  |
|  | LZD 71 mg/kg (7/7) bid | 4.06±0.09 | 2.47±0.10 | 5.38±0.18 | 0.10±0.14 |

**Supplemental Table 3.** Mean (± SD) values from complete blood counts obtained after 2 months of LZD treatment in infected BALB/c mice. Abbreviations: LZD, linezolid; qd, once a day dosing; qod, every other day dosing (M-W-F); bid, twice a day dosing.

| **Total Weekly Dose** | **Regimen** | **Hematocrit (%)** | **WBC (K/µL)** | **Platelet (K/µL)** |
| --- | --- | --- | --- | --- |
| **Control** | **Untreated** | 51.20±2.10 | 6.70±1.29 | 978.00±223.45 |
| **100 mg/kg** | **LZD 33 mg/kg (3/7) qod** | 41.85±2.05 | 8.24±0.17 | 1426.00±2.83 |
|  | **LZD 20 mg/kg (5/7) qd** | 42.60±1.49 | 8.08±3.18 | 1192.00±330.89 |
|  | **LZD 10 mg/kg (5/7) bid** | 39.77±0.05 | 6.40±0.41 | 1501.33±125.48 |
| **300 mg/kg** | **LZD 100 mg/kg (3/7) qod** | 40.63±2.40 | 7.60±1.98 | 1416.67±41.88 |
|  | **LZD 60 mg/kg (5/7) qd** | 35.17±7.27 | 7.25±1.24 | 1060.67±500.03 |
|  | **LZD 30 mg/kg (5/7) bid** | 35.17±3.16 | 7.48±0.30 | 1325.33±154.30 |
| **1000 mg/kg** | **LZD 333 mg/kg (3/7) qod** | 38.47±0.56 | 9.50±3.04 | 1479.67±299.27 |
|  | **LZD 200 mg/kg (5/7) qd** | 29.67±3.50 | 9.06±1.71 | 1432.00±351.07 |
|  | **LZD 100 mg/kg (5/7) bid** | 18.00±2.37 | 9.02±.064 | 1262.67±28.02 |

**Supplemental Figure 1.** Exposure-response relationships for LZD-induced anemia, as measured by (A) HCT and (B) RBC in infected BALB/c mice. Abbreviations: LZD, linezolid; HCT, hematocrit; RBC, red blood cells; C_min_, LZD minimum concentration; AUC, area under the concentration-time curve; C_max_, LZD maximum concentration.
